# Mobile resonance frequency breathing smartphone application to support recovery among people with opioid use disorder: Study protocol for feasibility study

**DOI:** 10.1371/journal.pone.0296278

**Published:** 2024-01-31

**Authors:** Fiona N. Conway, Heather Kane, Michele Dorsainvil, Patrick Kennedy, Jessica D. Cance

**Affiliations:** 1 Addiction Research Institute, Steve Hicks School of Social Work, The University of Texas at Austin, Austin, Texas, United States of America; 2 RTI International, Durham, North Carolina, United States of America; University of Maryland at College Park, UNITED STATES

## Abstract

**Background:**

Experiencing drug cravings is an aspect of substance use disorders that frequently compromises the recovery efforts of people who use drugs. Most treatment approaches that address drug cravings either involve cognitive strategies or medication. Few interventions directly address the physiological aspects of craving, such as increased heart rate. Previous research has demonstrated that slow-paced breathing may be effective in managing drug cravings by manipulating an individual’s heart rate. The purpose of this paper is to describe a study protocol for an intervention that offers resonance frequency breathing training for managing cravings via a smartphone application (app).

**Methods:**

This trial is registered in ClinicalTrials.gov (Identifier: NCT05830773). The intervention focuses on persons in recovery from opioid use disorder who receive services from the Texas Health and Human Service Commission Recovery Support Services division. Participants will be trained to use Camera Heart Rate Variability (CHRV), a resonance frequency breathing app. The CHRV app measures heart rate and the volumetric variations of blood circulation. When experiencing stress, anxiety, or cravings, participants will use the app to practice breathing exercises. Participants (N = 60) will also complete surveys at baseline, 4 weeks, and 8 weeks; the survey questions, covers demographic characteristics, personal trauma history, substance use experience, and utilization of substance use treatment services. The surveys will also include psychosocial measures of craving, stress, and anxiety to allow the study team to assess changes between baseline and study completion. Participants who complete the full 8-week intervention will be invited to participate in a 30-minute interview about their experience with the app. Interviews will provide details on implementation outcomes, including acceptability, appropriateness, and feasibility.

**Conclusion:**

Many evidence-based interventions for opioid use require interpersonal communication with individuals in one’s recovery network. However, individuals may be unable to engage others in their recovery network in the moments when they are experiencing cravings or stress- and anxiety-related triggers. Therefore, recovery support interventions that emphasize individual self-management of cravings, stress, and anxiety when they occur can empower individuals in recovery and enhance existing interventions.

## Introduction

The United States is in the midst of a drug overdose crisis. Provisional data from the Centers for Disease Control and Prevention (CDC) identifies over 105,000 overdose-related fatalities in the 12 months prior to November 2021, the highest number on record since CDC began tracking drug overdose mortality. Deaths, however, represent only a small proportion of the burden of substance use disorders (SUD); in 2020, over 21 million adults in the United States self-identified as being in recovery or as having recovered from problematic substance use [1].

SUD treatment and recovery has long focused on mitigating the factors that contribute to an individual’s substance use. Some behavioral health interventions and mutual aid programs, such as 12-step recovery programs, typically aim to strengthen the resolve of people who use drugs (PWUD) to avoid environmental triggers, which can be categorized into people, places, and things [2]. Many interventions also emphasize interpersonal communication with individuals in one’s recovery network as a strategy for coping with triggers. Addressing triggers is a critical part of SUD treatment: during the recovery process, cravings for alcohol, tobacco, and other drugs (ATOD) are often elicited by stress and anxiety that accompany encounters with triggers. Craving, conceptualized as a strong desire to use drugs [3], has psychological (e.g., mood changes) and physiological (e.g., increased heart rate) indicators [4]. These cravings can occur during times when individuals are not in an intervention or care setting, cannot reach others in their recovery network, or cannot avoid triggers. However, many existing interventions do not provide daily training for the self-management of cravings in the moment they occur. This is a critical gap in treatment, as an individual’s ability to manage their cravings is essential for long-term recovery.

Heart rate variability biofeedback (HRVB) is a promising intervention that can address this treatment gap by utilizing the body’s physiological mechanisms to manage craving [5–8]. It refers to a mind-body technique that uses paced breathing and visual and sound feedback to help individuals manage and gain control over involuntary body functions, such as blood pressure and blood flow. HRVB stimulates various homeostatic reflexes (such as vagus nerve activation) to induce relaxation [9]. Previous research has demonstrated that HRVB is a suitable supplemental intervention to traditional substance use treatment modalities [5,6,8,10,11]. A core component of HRVB is breathing at an individualized optimal rate (4.5 to 6.5 breaths per minute) that synchronizes heart rate with respiration [12]. Identifying the optimal breathing rate requires the use of sensors attached to the body to measure heart rate and respiration, as well as a visual monitor to display those results [13]. HRVB training traditionally occurs in an office or laboratory with the assistance of a trained technician. Although there has been an increase in the number of portable HRVB devices during the past decade, HRVB still requires the identification of an individualized breathing rate, a complex process requiring training that is not easily accessible to the general public [12].

Resonance frequency breathing is an approach that addresses this limitation; it requires breathing at a pace of six breaths per minute and produces similar results to breathing at optimal rates identified during HRVB. Although research supports HRVB as an adjunct to SUD treatment, little research has focused on the utility of self-administered resonance frequency breathing exercises via mobile technologies. The purpose of this paper is to describe a mobile resonance frequency breathing intervention, which offers resonance frequency breathing training for managing cravings via a smartphone application (app). This intervention allows individuals to use resonance frequency breathing during their everyday lives and in settings where they are more likely to be exposed to environmental craving triggers. Use of the app is intended to reduce the following outcomes: cravings, stress and anxiety.

## Materials and methods

**Fig 1:**
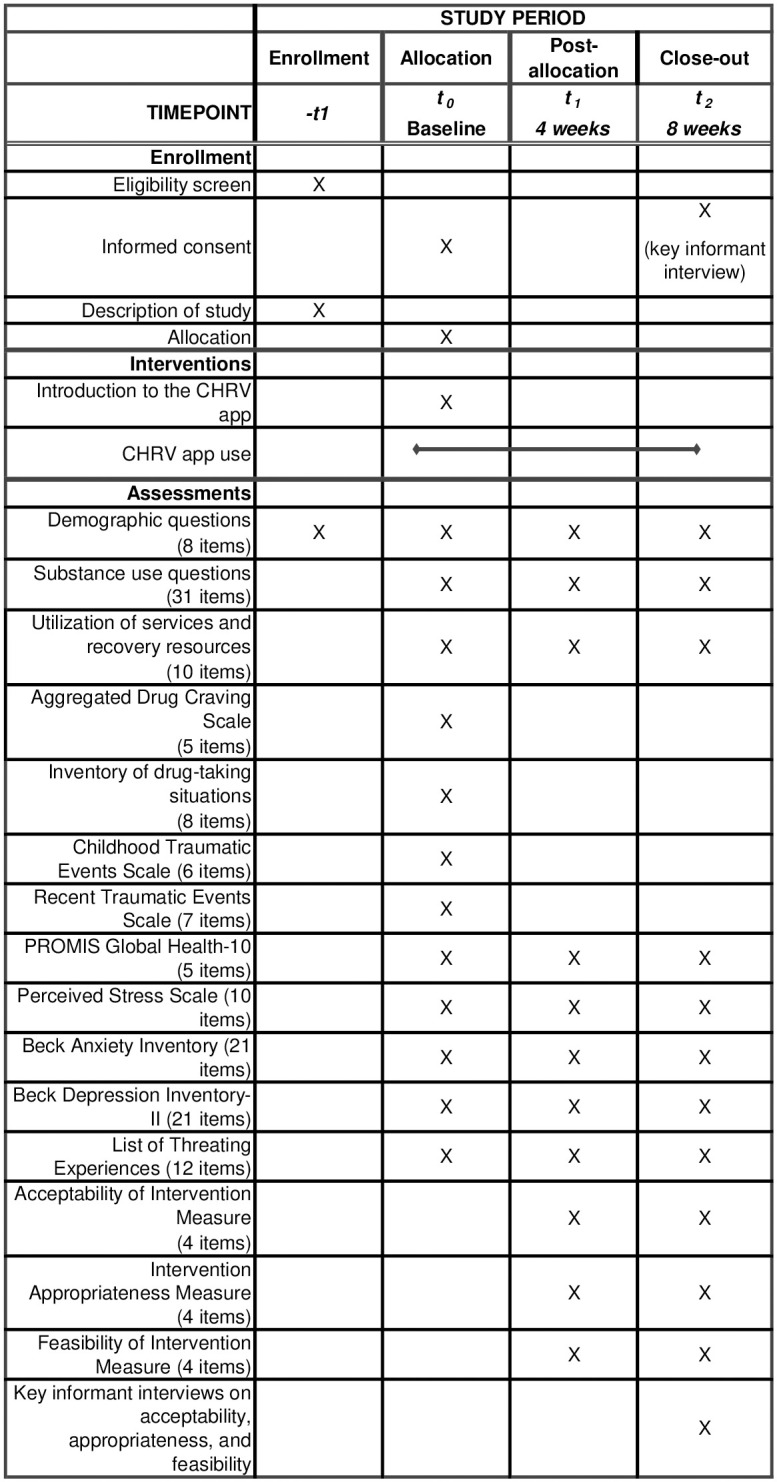
Schedule of enrollment, intervention, and assessments.

This study is part of the Texas Targeted Opioid Response (TTOR), public health initiative. The goal is to “address the opioid crisis by reducing unmet treatment need and opioid overdose-related deaths through its evidence-based programming” [14]. This protocol focuses on a resonance frequency breathing project to support recovery. Data collection began in March 2021 and will conclude in September 2023; the intervention will occur in Texas (United States). The University of Texas at Austin’s Institutional Review Board has reviewed and approved all study materials and procedures. The protocol is reported according to the SPIRIT guidelines [15] (S1 Checklist). The SPIRIT schedule of enrolment can be found in Fig 1.

### Participants

The intervention focuses on persons in recovery from SUD who receive services from the Texas Health and Human Service Commission (TXHHSC) Recovery Support Services division. To be eligible, participants need to have reached the legal age of majority (18 years of age or older), have the ability to read and speak in English, and have a history of alcohol or illicit drug use. History of alcohol or illicit drug use is determined by a participant’s status as recipients of recovery support services. Because the intervention involves use of a phone app and sending data on app use to the study team, participants also need access to a smartphone with a data plan. Exclusion criteria include the inability to provide consent and having suicidal thoughts or psychotic episodes. Individuals who could experience greater than minimal emotional distress prompted by the intervention surveys, which have questions pertaining to childhood trauma and substance use, can refuse to continue participation or skip those questions. Peer support specialists at the TXHHSC Recovery Support Division will offer counseling and supportive services to any individuals who experience distress because of the survey. The study team will also be trained to respond to individuals experiencing distress; they will recommend that the individual speak to a peer support specialist at TXHHSC and will provide information about community mental health resources. The study team will discontinue the study protocol for such individuals. The study team will provide honoraria (described below) to encourage participation in the intervention.

### Recruitment

Data will be collected using a convenience sampling method from individuals who are recovering from SUD and receive services at the TXHHSC Recovery Support Services division. Peer support specialists from TXHHSC Recovery Support Services division will assist the study team in recruiting participants for the project. The study team will train the peer support specialists on how to explain the study and invite individuals to participate. The peer support specialist will email the study team contact information (name, mail, phone number) for eligible individuals who meet the screening criteria. The study team will then contact the potential participant to schedule a phone conversation to discuss the study, answer questions, and review the informed consent agreement. After this conversation, the study team will assign a unique study ID to each person who wishes to participate and will send them an email request to complete an online survey that includes an informed consent document and questionnaire on demographic data (e.g., age, race/ethnicity, sex, height, weight); psychosocial measures of craving, stress, trauma history, depression, anxiety, and substance use; and other measures of behavioral and general health.

**Fig 2:**
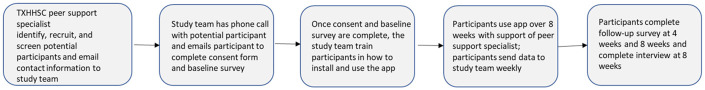
Recruitment and data collection procedures.

After an individual completes the informed consent and questionnaire, a study team member will contact them and identify a mutually agreeable time to help them install the breathing app and train them how to use it. The participant will be instructed on how to email data regarding date, time, and duration of app use to a secure study email address. A peer support specialist will hold regularly scheduled meetings independent from the trial to check in with participants, encourage use of the app, and provide assistance as needed. Fig 2 displays the recruitment and data collection procedures.

### Procedures

#### Study design

The program is a single group design with no control group. At baseline, participants will complete an online questionnaire that includes demographic information, substance use patterns, and validated psychosocial measures on stress, trauma history, craving, anxiety, depression, and other areas. At weeks 4 and 8, study team members will email participants links to shorter online questionnaires to complete. Fig 1 displays which items will appear on the surveys at each time point. Validated assessments in the survey include the Childhood Traumatic Events Scale [16], Recent Traumatic Events Scale [16], Inventory of Drug-Taking Situations [17], Aggregated Drug Craving Scale [18], PROMIS Global Health-10 [19], Perceived Stress Scale [20], Acceptability of Intervention Measure, Intervention Appropriateness Measure, Feasibility of Intervention Measures [21], Beck Anxiety Inventory [22], Beck Depression Inventory-II [23], and the List of Threatening Experiences. Participants will receive a $21 honorarium for completing each survey (total of $63 for completing all three surveys). Compensation for each survey will be provided within 1 week of completing that survey.

In addition to the surveys, participants will be instructed to email app usage data once a week to a secure study email address. The app collects the date, time, and duration of use, as well as the participant’s heart rate during use; the app will not collect any other data from the phone (e.g., calls, websites visited, content of text messages). App data is stored locally on the phone. The app does not have any backend or server and data is not transmitted to other servers. If the user deletes the app, all data is also deleted. Participants will be encouraged to continue to use the app at no charge after completing the study.

Participants share app data with the study team by choosing the “export” function, entering the study team email address, and selecting to send the email. Participants will receive compensation for the number of days they used the app ($2/day) for a total of up to $112 for 56 days of usage. Compensation will be sent to participants every 2 weeks.

Upon completion of the 8-week intervention period and of the final survey, the study team will send each participant an email invitation to do an interview about their experience with the app. Interviews will occur via phone or videoconference, should take no more than 60 minutes to complete, and will be audio-recorded and transcribed with participant permission. Participants will receive a $35 honorarium for completing the interview. Interview topics will center on the three main indicators of implementation success—appropriateness, feasibility, and acceptability [24]. Appropriateness questions will assess the alignment between the participants’ needs and the app functions. Feasibility questions will examine whether participants found the app easy and practical to use; acceptability questions will explore participant beliefs about the quality of the app and whether the app worked well for managing cravings, stress, and anxiety.

#### Data management and monitoring

Any participant who meets the eligibility criteria will be assigned a unique ID number. Their name and unique ID will be added to a master list and saved on a secured university ethics–approved storage application. The unique ID will be used to track all data related to the participant (e.g., the survey data, data on app usage, interview transcripts). Only study team members will have access to the data. Data will be destroyed within three of years of completion of the intervention. There are no plans to have a data monitoring committee for this study because it is a minimal risk study at the early stages of development.

#### Description of the intervention

This intervention uses the Camera Heart Rate Variability (CHRV) [25] smartphone application, a resonance breathing app that is available for download to iPhones and Android devices. The CHRV app uses photoplethysmography (PPG) technology to measure heart rate. PPG is a noninvasive technology that uses a light source (the phone’s flashlight) and a photodetector (the phone’s camera lens) at the surface of one’s skin to measure the volumetric variations of blood circulation [26]. The app also has a timer, which allows the participant to see how long they use the app in each session. The iPhone version of the app has a breathing pacer (a bar that moves up and down to show the participant when to inhale and exhale). The app also provides feedback on heart rate.

The study team will train peer support specialists to deliver the intervention to participants. The training will include an introduction to guided breathing theories and instruction on using the CHRV app. Once a participant has completed the consent and baseline survey, a peer support specialist will instruct them on how to download and use the app. Participants will be instructed to initiate a resonance breathing session for at least 5 minutes daily and whenever they experience cravings, feel like they are going to relapse, feel anxious or stressed, or just want to feel calm. The app does not send reminders to complete breathings exercises. The intervention is designed for on demand delivery of the breathing exercises. Participants can use the app at a quiet and private location of their choosing (e.g., home, worksite).

When participants in the intervention experience cravings, stress, and anxiety, they can use the app to initiate a resonance breathing session. During a breathing session, the app participants will cover their phone’s camera lens with a finger (the index finger is recommended) to allow the camera to detect their heart rate. A timer and graphical depiction of their heart rate in real time will automatically appear on the phone screen. Participants who use iPhones will also see a breathing pacer. Ideally, the breathing exercise will lower the participant’s heart rate and reduce physiological sensations of craving, stress, and anxiety. Peer support specialists will hold weekly meetings with participants to check in on participant progress using the app and to address any questions.

### Analysis plan

#### Project aims

**Fig 3:**
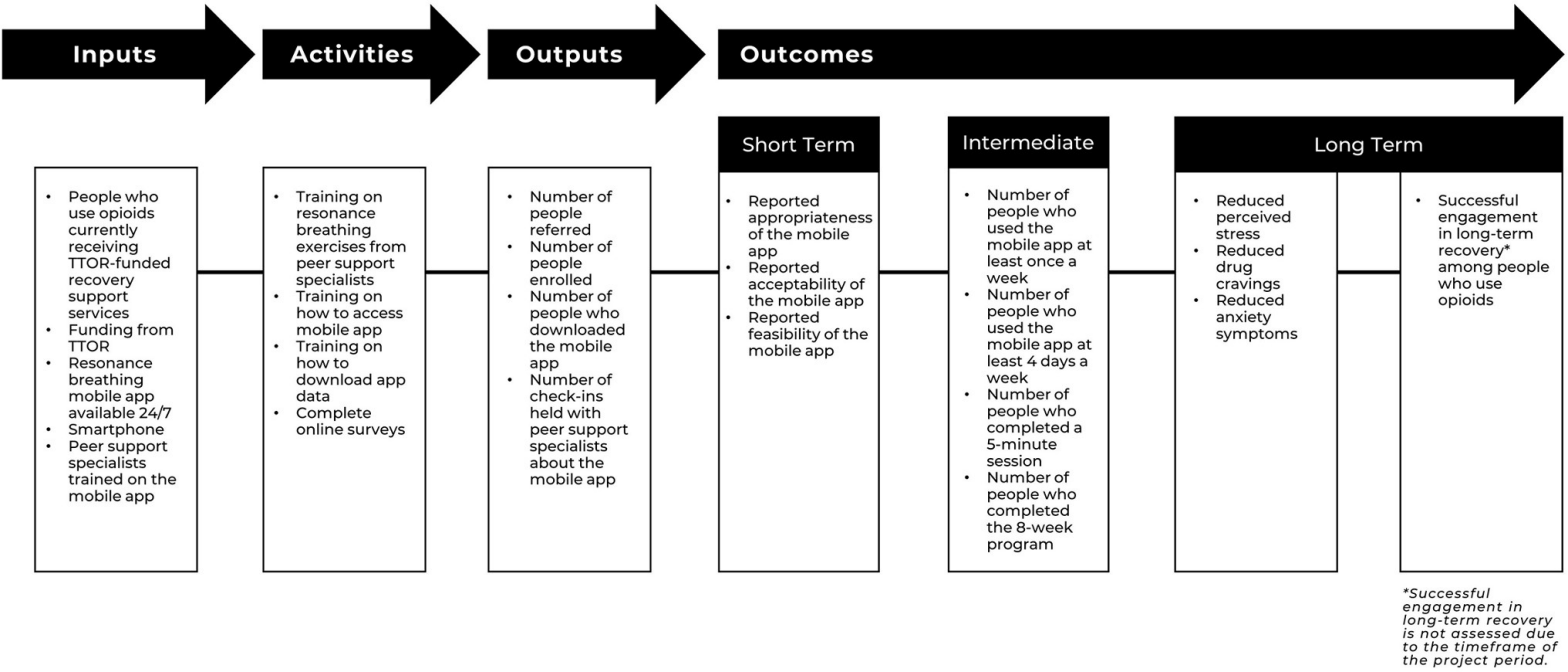
Intervention logic model.

The goals of this study include an enrollment of 60 participants and a 90% rate of participants using the app at least once daily for an average of 5 minutes per session. Fig 3 displays the logic model for the intervention activities and intended outcomes. The study team will assess primary and secondary implementation outcomes through statistical analyses of the survey responses. They will also assess implementation outcomes through qualitative analysis of interview data.

For the primary outcomes, the team will assess whether various durations of app use are associated with changes in cravings, stress, and anxiety. Repeated regression analysis will be used to account for individual variability. The study team will examine whether patterns of usage differ across participants and, if so, whether these patterns are associated with outcomes. In addition to the regression analyses, the team will use descriptive statistics to examine whether (1) participants found the app appropriate for their goals and needs, (2) it was easy and practical to use, and (3) how it helped them manage their cravings.

#### Screening questions

Participants will be screened to ensure that they are 18 years of age or older, have a history of alcohol and illicit drug use, have access to a smartphone with a data plan, and can read and speak in English.

#### Primary outcomes

The study team will examine the effects of the app use on several primary outcomes: (1) cravings, (2) stress, and (3) anxiety. For all measures, the interest is seeing reductions from baseline to final assessment.

*Cravings*. Using the Aggregated Drug Craving Scale, [18] the team will assess cravings over the past month. The scale includes five validated questions about the frequency, intensity, and duration of cravings; the ability to resist cravings; and an overall assessment of cravings measured on a scale from 0 to 6. Lower scores indicate less craving. Craving will be included in the baseline, 4-week, and 8-week online surveys.

*Stress*. The team will use the Perceived Stress Scale [20] to assess perceived stress. This scale has 10 items and asks respondents to rate how often from 0 (never) to 4 (very often) they have experienced symptoms of stress (e.g., feelings of loss of control, inability to cope) during the past month. Lower scores indicate less stress. Stress will be assessed in the baseline, 4-week, and 8-week online surveys.

*Anxiety*. The team will use the Beck Anxiety Inventory [22] to assess anxiety. This inventory consists of a list of 21 symptoms of anxiety (e.g., numbness, tingling, feeling hot) and the extent to which an individual has experienced each symptom over the past month (not at all, mildly, moderately, or severely). Each item has a range of severity scores from 0 (not at all) to 4 (severely). Lower scores indicate less anxiety. Anxiety will be assessed in the baseline, 4-week, and 8-week online surveys.

#### Secondary implementation outcomes

The study team will assess intervention implementation effectiveness using the Acceptability of Intervention, Intervention Appropriateness, and Feasibility of Intervention measures. [21] Each measure includes four questions that assess acceptability (e.g., the app meets my approval), appropriateness (e.g., the app is suitable), and feasibility (e.g., the app seems easy to use). The items are assessed using a Likert scale with five options (completely disagree, disagree, neither agree nor disagree, agree, and completely agree). For each measure, we will calculate the mean across the four items.

*Acceptability*. Acceptability refers to participant satisfaction with the app. Questions to capture acceptability include whether the participant used the app, what encouraged (or discouraged) their use of the app, and their assessment of the app’s quality and functionality.

*Appropriateness*. Appropriateness is the participants’ assessments of the app’s utility in managing cravings, stress, and anxiety. Questions include what goals or needs encouraged the individual to participate in the intervention, whether the app helped the participant meet those goals or needs, what features of the app addressed their goals or needs, and what features should be added to help address participant goals or needs.

*Feasibility*. Feasibility entails participant assessment of whether the app was easy and practical to use. Feasibility questions include how technologically complicated the participant found the app, what level of support (e.g., training or assistance) the participant needed to set up and use the app, and whether it was practical for the participant to use the app in their day-to-day life.

To contextualize and triangulate with the results of the quantitative implementation measures, the team will also explore the three indicators of implementation success in post-completion participant interviews. The team will use a semi-structured interview guide to ask participants open-ended questions.

#### Planned analyses

*Quantitative analysis*. The study team will link each participant’s survey responses and app use data via their unique study ID numbers in a single master data file. After cleaning the data (i.e., checking for missing data and outliers), the team will run descriptive statistics. We will use a mixed modeling approach (e.g., PROC GLIMMIX in SAS) to assess changes in the outcomes over time. We will also look at differences in the outcomes by app usage. The study will enroll 60 study participants. Using mixed-effects regression models our power analysis, simulated with 10,000 replications, shows that we will have sufficient power (0.86) to detect a medium effect size (0.4) of changes in clinical outcomes (cravings, stress, and anxiety). Repeated measures analysis of variance will be conducted to determine within-person changes in cravings, stress, and anxiety during the project period. All quantitative statistical analyses will be conducted using SAS 9.4 software.

*Qualitative analysis*. Interview data will be collected via phone or videoconference and will be audio-recorded and transcribed with participant permission. All data will be stored on secure servers accessible by study team members only. Interview transcripts will be entered into qualitative data management software NVivo 20 and coded using a codebook that focuses on implementation outcomes (acceptability, appropriateness, and feasibility). A second coder will assess reliability by independently coding a subset of the transcripts and reviewing intercoder reliability. Upon achieving a minimum of a.7 Cohen’s kappa coefficient [27] for agreement, the team will run code reports and apply an inductive approach [28] to identify themes within each implementation outcome. Themes will be documented in an analytic matrix [29].

## Discussion

Many evidence-based interventions for opioid use require interpersonal communication with individuals in one’s recovery network. However, individuals may be unable to engage others in their recovery network when they experience cravings or stress- and anxiety-related triggers. This study will assess the feasibility of implementing an in-the-moment intervention (i.e., using a resonance breathing app) among persons who are in recovery, as well as assess preliminary outcomes related to consistent app usage. The study will use a convenience sample, which limits conclusions about generalizability of potential findings related to the clinical outcomes. However, this is a pilot trial focusing on the feasibility of implementing the intervention and can provide preliminary information to (1) inform whether and how the intervention should be revised and (2) demonstrate preliminary outcomes, which can affect whether and how to pursue a larger trial. Further, power to detect changes in the outcomes of interest will depend on intervention enrollment and participants’ ongoing willingness to submit data over an 8-week period. Weekly engagement with a peer support specialist may encourage participants to consistently submit data; additionally, should app use be less frequent than hoped, interviews with participants may help identify the barriers to app use (i.e., what made the app less appropriate, feasible, or acceptable to participants). Recovery support interventions that emphasize the individual management of cravings, stress, and anxiety in the moments they occur can empower individuals in recovery and can enhance existing interventions. Participants who use the resonance breathing app and practice resonance breathing may experience reductions in their stress, craving, and anxiety that may ultimately enhance their ability to maintain successful recovery.

## Supporting information

S1 Checklist SPIRIT 2013 checklist: Recommended items to address in a clinical trial protocol and related documents*. (PDF)

S1 File (PDF)
