# Supplementary material for: Mobile resonance frequency breathing smartphone application to support recovery among people with opioid use disorder: Study protocol for feasibility study
Source: PLoS One. 2024 Jan 31;19(1):e0296278. doi: 10.1371/journal.pone.0296278 (PMC10829996; doi:10.1371/journal.pone.0296278)
Supplement: S1 File — (PDF) [file pone.0296278.s002.pdf]

FORM: IRB Proposal - Standard Submission	
NUMBER	VERSION DATE
HRP-UT901	8/3/2020

GENERAL STUDY INFORMATION

Use for greater than minimal risk studies and minimal risk studies that fit into one or more expedited categories (see Section 5.3 of our Policies & Procedures for details regarding expedited research).

Do NOT submit this form if the study will qualify for exempt review, instead submit HRP-UT902 IRB Proposal – Exempt Submission Form found in the document Library.

If you are only using secondary data that will not be initially collected solely for this research project, use HRP-UT903 Template IRB Proposal Secondary Use form instead.

For studies following a multi-center or sponsor protocol, please use this guidance to assist in your completion of this form.

For questions regarding definitions, policies, or terms referenced below see the policies and procedures manual.

Please note, Word online does not support Word checkboxes. Please download the file and use your desktop version of Microsoft Word.

1 Review Type (Choose one)

*Click on the check box (or double click and type an "X" if using Google Docs) the **one** review type that applies.*

Please note: Expedited Review does not refer to the timeliness of the review of your protocol, but specific categories of research defined by ORHP. If you would like help determining which type of review is most appropriate for your study please contact the Office of Research Support and Compliance: https://research.utexas.edu/ors/about-ors/contact-us/.

a ☐ **Full Board Review – Greater than Minimal Risk Research**

b ☒ **Expedited Review – Minimal Risk Research**

2 Research Hypothesis

To input text, click in the light grey area below.

People in recovery from hazardous drug and alcohol use will experience a decrease in perceived stress, drug craving, anxiety, and depression through use of a paced breathing biofeedback intervention via the Camera Heart Rate Variability (CHRV) app; a mobile phone software application.

3 Study Background

To input text, click in the light grey area below.

Most evidence-based treatments for substance use disorders (SUD) require face-to-face interactions with individuals who comprise a recovery support network. However, in the moment relapse occurs, people in recovery are often not in the presence of these trusted individuals. This project aims to address this issue by providing people in recovery with a CHRV App that can be added to phones and other mobile devices to help them manage cravings and stress triggers at the specific moments they feel compelled to use. The connection between our minds and our bodies works in both directions. Psychological distress can cause psychosomatic symptoms in the body (e.g., stress is related to multiple chronic health conditions), but we can also use our bodies to affect our psychology (e.g., exercise can improve mood by releasing endorphins). A similar scientific theory is the foundation of controlled breathing interventions for stress and drug cravings. Psychological distress can disrupt the balance of our Autonomic Nervous System (which is responsible for our fight or flight response to threat), but breathing exercises can restore balance and decrease stress. The goal of this study is to test the feasibility, utility, and efficacy of the CHRV app.

4 Design and Methodology

Provide information regarding study design or data collection methodologies. Details regarding protocol specific research procedures will be discussed in a later section.

To input text, click in the light grey area below.

This is a longitudinal study. Each participant will be involved in an 8-week testing of the CHRV smartphone app. The CHRV app computes measures heart rate with the phone's camera flashlight by using Photoplethysmography (PPG) technology. PPG is a non-invasive technology that uses a light source (the camera flashlight) and a photodetector at the surface of skin to measure the volumetric variations of blood circulation. The app also has a timer (to allow the participant to see how long they are using the app for each session), and a breathing pacer (a bar that moves up and down to show the participant when to inhale and exhale). When the participant starts a breathing session with the app they cover the flashlight of their phone with their finger to allow the camera to detect their heart rate. The breathing pacer and a time display will automatically appear and the participant will see their heart rate as they do the breathing exercise. The funding agency (Texas Health and Human Services) has a recovery support services division. An employee of the division will assist the principal investigator in recruiting participants for the study. Data regarding the date, time, and duration of use of the CHRV app will be emailed by the participant to the secure email server at the university. Participants will also complete three online questionnaires that

include demographic information, substance use patterns, and validated psychosocial measures including of stress, trauma history, drug craving, anxiety, and depression. Participants will also complete a 30-minute follow-up interview remotely via video or phone conference. The funding agency (Texas Health and Human Services) sponsors multiple programs (THHSP) dedicated to prevention, treatment, and recovery for PERs. Participants in the study will be recruited by referrals from THHSP staff and flyers posted at the program locations.

5 Data Analysis

Describe the data analysis plan, including any statistical procedures or power analysis.

To input text, click in the light grey area below.

Quantitative and qualitative data will be collected. Repeated measures analysis of variance will be conducted to determine within-person changes in stress, anxiety, and depression during the project period. Mixed-effects regression models will be used to examine the relationship between various psychosocial measures and the CHRV app utilization. All quantitative statistical analysis will be conducted using SAS 9.4 software. Thematic analysis of the qualitative data will be conducted. Thematic analysis of the qualitative data will be conducted using NVivo 1.6 software.

STUDY ELEMENT IDENTIFICATION

6 Study Elements

Click on the check box (or double click and type an "X" if using Google Docs) each procedure included in your study.

A full description of all study procedures should be provided in the Procedures (Details) section below and/or the applicable supplement form.

☐	**Bio-specimens**	☐	**Biometrics**	☐	**Registry or Repository**
☐	**Focus Group**	☐	**Genetic Analysis**	☐	**Genomic Data Sharing**
☐	**International Research**	☒	**Interview/Survey**	☐	**MRI**
☐	**Protected Health Information**	☐	**Observation**	☐	**Record Review**
☐	**Sensors**	☐	**Sensors**		**Video/Audio Recording**

(Externally Placed)	(Inserted)	

☐ X-Ray

7 Study Intervention

Click on the check box (or double click and type an "X" if using Google Docs) if you will implement any of the following interventions.

A full description of all study interventions should be provided in the Procedures (Details) section below and/or the applicable supplement form.

☒ Behavioral ☐ Device ☐ Drug/Biologic

8 Clinical Trial

Click on the following check box (or double click and type an "X" if using Google Docs) if the research meets the below definition of a clinical trial.

☐ **This study meets the definition of a clinical trial according to clinical trials.gov in that it involves one or more human subjects who are prospectively assigned to one or more interventions (which may include placebo or other control) to evaluate the effects of those interventions on health-related biomedical or behavioral outcomes.**

9 Additional Oversight

Click on the check box (or double click and type an "X" if using Google Docs) each activity that requires oversight from additional UT committees.

☐ Biohazards, Recombinant DNA, or Gene Transfer

☐ Energy introduced to the subject (electrical, magnetic, light)

☐ Human embryonic, human induced pluripotent, or human totipotent stem cells; or human gametes or embryos

☐ Radiation exposure without direct clinical benefit

10 Alternatives to Participation in This Study

To input text, click in the light grey area below.

The alternative is not to participate in the study. If an individual chooses not to participate, the study protocol with not be administered.

STUDY PROCEDURE DESCRIPTION

11 Procedure Description

Describe all study procedures, including a step-by-step outline of what participants will be asked to do or how data will be used. Be sure to describe all of the following in detail, as applicable:

- Provide a description of all research procedures being performed and when they are performed, in sequential order.
- All research measures/tests that will be used and state if questions or measures are standardized or published (upload copies of all surveys, scripts and data collection forms)
- Secondary data or specimens that will be obtained, how they will be collected, and how they will be used
- Where each activity will take place, the duration of each, and who will perform each activity
- Include time commitment of participants

To input text, click in the light grey area below.

During the time-period of restricted limitations on in-person interactions (i.e., during the COVID-19 pandemic) research personnel will conduct all consent, data collection, and intervention activities with participants using remote procedures (i.e., video conferencing or phone calls). The funding agency, Texas Health and Human Services Commission (TXHHSC), has a recovery support services division. Employees of the division (TEs) will assist the research personnel in identifying participants for the study. The TEs will also be trained by research personnel on how to assist participants with installing the App on their phones and provide instructions on how to use the app.

The app only collects information about app usage (heart rate and days and times of the app use). The app does not collect any other data from the phone (e.g., calls, websites visited, content of text messages, etc.). This will be explained to the participant during the consent process. No one other person or entity owns the data. The data is stored only locally on the phone. The app does not have any backend / server, and data is not transmitted anywhere. Data collected through the App will be protected, and no one outside the research team will have access to it. If the user deletes the app, all data is also deleted (and cannot be retrieved in any way).

The TEs will not be involved in answering questions about the study other than to invite people to participate. They will not be obtaining consent or administering measures.

1. Research personnel will meet with TEs (video conferencing or phone calls) to explain the study and discuss how to invite people to participate. They will direct people who are interested to contact research personnel to answer questions about the study.
2. The TEs will email contact information (name, email, phone number) of people who would like to participate to the research personnel.
3. Research personnel will contact the people who express interest to have a phone conversation about the study to answer their questions and review the informed consent agreement.
4. Research personnel will send people who agree to participate an email request to complete an online survey that includes an informed consent document and questionnaire that includes demographic data (age, race/ethnicity, sex, height, weight, etc.) and psychosocial measures of perceived stress, trauma history, depression, anxiety, substance use, and other measures.
5. When the informed consent and questionnaire are complete, TEs will contact the participants and identify a mutually agreeable time to help them install the App and train them how to use it. The participant will be instructed to email data regarding date, time, and duration of use to a secure UT Austin email address.
6. Research personnel will send participants and email that instructs them to complete two additional surveys at the 4-week and 8-week point of the study.
7. Upon conclusion of the 8-week program, research personnel will contact the participant to schedule a date and time for their interview.
8. Research personnel will conduct a 30-minute follow-up interview remotely via video or phone conference using an interview guide. The interview will be video or audio recorded.

SUBJECT POPULATION

12 Protected Subject Populations

Click on the check box (or double click and type an "X" if using Google Docs) each population, if they are specifically studied for this research.

☐ Active military personnel	☐ Children	☐ Decisionally impaired adults
☐ Emancipated minors	☐ Fetuses	☐ Individuals with limited English proficiency
☐ Neonates	☐ Pregnant Woman	☐ Prisoners
☐ UT Students	☐ UT or Seton Staff/Employees	

13* Research Participant Information

Describe the research population.
**For multiple research populations (e.g., teachers, students, and parents), copy this section as necessary to describe your population.*

a Participant Group Name

To input text, click in the light grey area below.

People who engage or are in recovery from hazardous drug and alcohol use

b Minimum Age

To input text, click in the light grey area below.

18 years-old

c Maximum Age

To input text, click in the light grey area below.

100 years-old

d Inclusion Criteria

To input text, click in the light grey area below.

Must be legal age of majority (18 +), history of alcohol and illicit drug use, access to a phone with a data plan, ability to read and speak in English.

e Exclusion Criteria

To input text, click in the light grey area below.

Inability to provide consent, actively suicidal, or psychotic.

f Additional Population Information

To input text, click in the light grey area below.

People in recovery who receive services from the Texas Health and Human Services Commission (TXHHSC) Recovery Support Services division.

14 Total Sample Size

To input text, click in the light grey area below.

N = 60

15 Sample size rationale

To input text, click in the light grey area below.

60 participants are sufficient to power a repeated regression analysis of change in stress, anxiety, and depression measured at three time points and to conduct thematic analysis of interview data.

SCREENING AND RECRUITMENT

16 Identification and Screening

Click on the check box (or double click and type an "X" if using Google Docs) if true.

☐ **This study involves obtaining information or biospecimens for the purpose of screening, recruiting or determining eligibility of prospective subjects prior to informed consent by either:**

1. Oral or written communication with the prospective subject or LAR
2. By accessing records containing identifiable private information or stored identifiable biospecimens.

17 Identification and/or Screening Procedures

Describe the identification and/or screening procedures below.
To input text, click in the light grey area below.

TXHHSC employees (TEs) will identify potential participants from among their clientele. Potential participants will be contacted by phone, email, or text by a TE and will complete a short (5-10 minutes) screening by phone to ensure they meet eligibility criteria.

18 Recruitment Overview

Click on the check box (or double click and type an "X" if using Google Docs) all recruitment methods utilized for this research.

☒	**E-mail**	☐	**Flyer**
☐	**In-Person**	☐	**Letter**

☐	Social Media	☐	Research Pool
☒	Telephone/Text	☐	Snowball Sampling
☐	Web-post	☒	Word of Mouth

19 **Describe the recruitment process, including where recruitment will take place.**

Describe the recruitment procedures below.
To input text, click in the light grey area below.

If the potential participant meets the screening criteria the TXHHSC employees will provide them with study information, and participants' rights and responsibilities as per the informed consent document by phone. They will be asked if they are interested in participating in the study; if they agree each participant will be assigned a unique ID number and asked for an email address. Our research team will send an email to the participant with a link to complete the informed consent form and the first of three online surveys.

OBTAINING INFORMED CONSENT

20 **Consent Overview**

Click on the check box (or double click and type an "X" if using Google Docs) all applicable items.

☐	Obtaining Written Informed Consent	☒	Requesting a Waiver of Documentation of Informed Consent
☐	Requesting a Waiver of Informed Consent	☐	Requesting an Alteration of the Required Elements of Informed Consent
☐	Obtaining Child Assent	☐	Obtain Consent Using a Short Form with a Witness

21 **Consent and Assent Processes**

Provide a detailed description of the consent process including who will obtain consent, where, and when consent will occur in such a manner that participants have sufficient time for adequate consideration.

To input text, click in the light grey area below.

Participants will have two opportunities to consider their consent decision: 1) during the screening process the TXHHSC employees will verbally review the informed consent document with the participant, 2) the first item in the survey will be the informed consent document. Participants will have an opportunity to read the document. The rest of the survey questions will be seen only if the participant chooses "I agree" for the informed consent.

22 Consent and Translation

Click on the check box (or double click and type an "X" if using Google Docs) to indicate that consent will be translated.

☐ **The study population will likely include participants whose limited English speaking status requires translation of the consent form.**

Translation Process

Click on the check box (or double click and type an "X" if using Google Docs) that best describes the translation process, either 21 or 22.

23 ☐ **The consent documents will be translated by a certified translator.**

24 ☐ **A non-certified translator will translate the consent documents.**

If selected, complete the next two questions below.

i **Describe the translator's qualifications**

To input text, click in the light grey area below.

ii ☐ **Another individual will confirm that the translation is accurate and appropriate.**

Waiver of Documentation of Informed Consent

To approve a waiver of documentation of informed consent, one of the following options below must be justified by the researcher.

Only complete the sections below if requesting a waiver of documentation of informed consent. *If not requesting a waiver of documentation of consent, skip to 27.*

Please choose one waiver option and provide additional information as prompted. The Office of Research Support and Compliance recommends using Waiver Option 2 in most cases.

25 Waiver Option 1

Provide confirmation for the following criteria and follow the additional instructions.

Additional Instructions:
1. Include this choice in the informed consent form.
2. Articulate the destruction process for signed consent forms in the privacy and confidentiality section.

Click on the check box (or double click and type an "X" if using Google Docs).

a ☐ **The only record linking the subject and the research would be the consent document.**

b ☐ **The principal risk would be potential harm resulting from a breach of confidentiality.**

c ☐ **Each subject will be asked whether the subject wants documentation linking the subject with the research, and the subject's wishes will govern.**

26 Waiver Option 2

Provide confirmation for the following criteria and follow the additional instructions.

Click on the check box (or double click and type an "X" if using Google Docs).

a ☒ **The study is minimal risk.**

b ☒ **Written consent would not be required outside the research context.**

27 Waiver Option 3

Provide confirmation for the following criteria and provide additional information as requested.

Click on the check box (or double click and type an "X" if using Google Docs).

a ☐ **The subjects or legally authorized representatives are members of a distinct cultural group or community in which signing forms is not the norm**

b **Describe the cultural group or community.**
To input text, click in the light grey area below

c ☐ **The research presents no more than minimal risk of harm to subjects.**

d ☐ **There is an appropriate alternative mechanism for documenting that informed consent was obtained.**

e **Describe mechanism for documenting that informed consent was obtained**

To input text, click in the light grey area below

Recruits will choose an “I agree” or “I decline” option after reading the informed consent text via the Qualtrics electronic survey link. If a participants chooses “I agree” the response will be saved and is available for data download to UT Box (a secure cloud server).

Waiver or Alteration of Informed Consent

To approve a waiver or alteration of informed consent all of the following criteria below must be justified by the researcher.

Only complete the sections below if requesting a waiver of informed consent. *If not requesting a waiver or alteration of consent, skip to 31.*

28 **The research involves no more than minimal risk to the subjects.**

To input text, click in the light grey area below

29 **The waiver or alteration will not adversely affect the rights and welfare of the subjects.**

To input text, click in the light grey area below

30 **The research could not practicably be carried out without the waiver or alteration (it is impracticable to perform the research if obtaining informed consent is required and not just impracticable to obtain consent).**

To input text, click in the light grey area below

31 **If the research involves using identifiable private information or identifiable biospecimens, the research could not practicably be carried out without using such information or biospecimens in an identifiable format.**

To input text, click in the light grey area below.

Deception and Debriefing

Only complete the sections below if requesting an alteration of informed consent that involves deceiving research participants. *If this study does not involve deception, skip to 35.*

See IRB Policies and Procedures Section 15 for a description of deception.

Click on the check box (or double click and type an "X" if using Google Docs).

32 ☐ **It is appropriate to provide additional pertinent information to the subject after research activities are complete (e.g., the researcher needed to deceive to subject to the nature of the study).**

33 ☐ **Research participants will have the opportunity to withdrawal their data during the debriefing.**

34 **Describe the nature of deception and why it is necessary to conduct the research.**

To input text, click in the light grey area below.

35 **Describe debriefing procedures.**

To input text, click in the light grey area below.

BENEFITS

36 **Benefits to Society**

Describe the scientific and societal benefit(s) below.

To input text, click in the light grey area below.

According to the National Survey on Drug Use and Health (NSDUH), 19.7 million Americans (aged 12 and older) battled a substance use disorder in 2017. Substance use disorders not only damages the lives of individuals, but also their loved ones and communities. The breathing biofeedback intervention (CHRV app) will help people who use drugs to reduce their cravings, stress, anxiety, and depression which will benefit everyone connected to them and society in general.

Benefits to Participants

Click on the applicable check box (or double click and type an "X" if using Google Docs).

37 ☐ **There is no anticipated direct benefit to participants.**

38 ☒ **There are anticipated benefits to participants.**

39 **If applicable, describe the potential direct benefits to participants.**

To input text, click in the light grey area below.

Participants who use the CHRV app by practicing slow-paced breathing may experience reductions in their perceived stress, drug craving, depression and anxiety.

RISKS

40 **Describe the risks associated with each activity in this research**

To input text, click in the light grey area below.

Participants may experience feelings of discomfort or emotional distress while completing the questionnaire that prompts them to self-report their stress, trauma history, substance use, anxiety, and depression. These pose minimal risks in that they are no greater than those the participants would encounter in their daily lives (e.g., talking with a friend about these issues) or during routine visits for healthcare treatment (e.g., completing forms at a doctor's office).

41 **Describe how each risk is mitigated/minimized.**

To input text, click in the light grey area below.

Subjective discomfort: any distress will be minimized by assurances that participant responses are confidential, that they can refuse to answer any particular question they do not want to answer, and that they are free to withdraw from the study at any time without penalty.

Data Safety Monitoring

For additional information regarding data safety monitoring boards and data safety monitoring plans, please see Section 21 of our Policies and Procedures.

Click on the check box (or double click and type an "X" if using Google Docs).

42 ☒ **This study is minimal risk and does not require a Data Safety Monitoring Plan (DSMP) or a Data Safety Monitoring Board (DMSB).**

43 ☐ **This study does not have a Data Safety Monitoring Board, but researchers have an internal plan/policy to monitor for safety.**

Complete Data Safety Monitoring Details (44-51).

44 ☐ **This study has a Data Safety Monitoring Board (DSMB).**

Complete Data Safety Monitoring Details (44-51) or upload this study's Data Safety Monitoring Board's charter.

Data Safety Monitoring (Details)

45 **How is safety information collected?**

To input text, click in the light grey area below.

46 **When will safety data collection start (for each participant or for the whole study, as applicable)?**

To input text, click in the light grey area below.

47 **How frequently will safety data be collected?**

To input text, click in the light grey area below.

48 **Who will review the data for safety?**

To input text, click in the light grey area below.

49 **How frequently will data be monitored for safety concerns?**

To input text, click in the light grey area below.

50 **What data will be reviewed?**

To input text, click in the light grey area below.

51 **State the frequency or periodicity of the review of cumulative data?**

To input text, click in the light grey area below.

52 **State any conditions that would trigger an immediate suspension of the research.**

To input text, click in the light grey area below.

Retraction of financial support from the funding agency.

Early Withdrawal

Only complete this section if there are planned conditions under which a participant will be withdrawn from the study. *If not applicable, skip to 56.*

Include this information in your consent form.

53 **List the criteria for withdrawing individual participants from the study (e.g., safety or toxicity concerns, emotional distress, inability to comply with the protocol, or requirements from study sponsor).**

To input text, click in the light grey area below.

Greater than minimal emotional distress prompted by completing the online surveys.

54 **Describe any necessary procedures for ensuring the safety of a participant who has withdrawn early.**

To input text, click in the light grey area below.

All participants are clients at the Texas Health and Human Services Commission (TXHHSC) recovery support services division. If a participant reports greater than minimal emotional distress they will most likely report it to their contact at TXHHSC. The employees at TXHHSC (TEs) are peer recovery support specialists who are trained and certified by the state to counsel people in recovery who are experiencing emotional distress. If a TE reports, to our research personnel, that a participant is experiencing greater than minimal emotional distress we will discontinue the study protocol for the participant. All UT research personnel on this study are graduates from a masters-level or higher clinical social work or psychology degree program, currently are enrolled in such program, or supervised by a PhD level mental health specialist. Before beginning work on this study, UT study personnel will be trained on how to respond to participants who verbalize greater than minimal feelings of emotional distress including: 1) asking the participant to share about what is making them feel uncomfortable, 2) providing them feedback on why this may be happening (e.g., answering questions about alcohol use may make them realize that they are

uncomfortable with their frequency of alcohol use), and 3) giving the participant the option to discontinue their participation. If the participant is not in immediate danger to themselves we will encourage them to speak with their peer recovery specialist at TXHHS, but we will NOT violate their confidentially by discussing what they shared with us with anyone at TXHHS. We will also provide them contact information to other community mental health providers. If the participant is an immediate danger to themselves or others the research team member will contact the local mobile health crisis center and/or police.

55 **Describe any pre-specified criteria for stopping or changing the study protocol due to safety concerns.**

To input text, click in the light grey area below.

If the participant is an immediate danger to themselves or others the research team member will stop the study protocol and contact the local mobile health crisis center and/or police.

REQUIRED DISCLOSURES

Required Consent Disclosures

Identify each element below that may require additional information to be disclosed in the consent form.

Click on the check box (or double click and type an "X" if using Google Docs).

56 ☐ **It is reasonable that researchers could discover or suspect child or elder abuse.**

57 ☐ **It is reasonable that researchers could learn of an incident that could require reporting under Title IX.**

58 ☐ **It is reasonable that researchers could discover incidental findings or other information of medical interest about a participant's previously unknown condition.**

59 **Articulate methods for addressing and reporting incidental findings, if applicable.**

To input text, click in the light grey area below.

60 **Privacy**

Describe how you will protect the identity and privacy of study participants during each phase of research. Privacy focuses on the individual participants rather than data. In this section, researchers should focus on issues such as where research activities take place and how participant involvement is protected from non-participants.

Describe methods to ensure participants' privacy during identification, recruitment, screening, the consent process, the conduct of the study, and dissemination of data.

To input text, click in the light grey area below.

Identification, recruitment, and screening: Texas Health and Human Services Commission (TXHHSC) employees (TEs) will identify, recruit, and screen the participants for this study. When a participant is identified, the TEs will provide the UT research personnel with their contact information (first name, last name, mobile phone number, and email address). We will create an excel document to use as a contact sheet. The privacy of the participants contact information will be ensured by being electronically stored on UT Box (an application approved by UT Austin for secure data storage).

The consent process and conduct of the study: The study will be conducted virtually. Participants will provide consent by selecting the "I agree" option after reading the consent form that will be embedded in the online survey on the UT approved secure Qualtrics electronic survey delivery software.

Dissemination of data: Only de-identified data will be disseminated in aggregate.

Confidentiality and Data Security Plan

Click on the check box (or double click and type an "X" if using Google Docs) that best describes the confidentiality and data security plan and provide additional details regarding how you will protect the confidentiality of data or address confidentiality concerns.

61 ☒ **Identifiers will be coded to protect confidentiality.**

61a **If true, state how data is coded and where identifiers are stored.**

To input text, click in the light grey area below.

Initial pre-screening will be conducted by phone by a Texas Health and Human Services Commission (TXHHSC) employee (TE). If the participant meets the

eligibility criteria and accepts the invitation to join the study the TE will email their name and contact information to a research team member. The participant will be assigned a unique ID number. Their name and unique ID will be added to a master list and saved as an excel document on UT box (an application approved by UT Austin for secure data storage). The unique ID will be used to track all data related to the participant (e.g., the survey data and data on App usage). All data will be stored on UT box.

62 ☒ **Identifiable data will be destroyed.**

62a **If true, describe destruction plan and timeline**

To input text, click in the light grey area below.

After three years the list that matches participants names to their Unique ID will be permanently deleted from UT Box. This will be the only link between participants and their data.

63 ☐ **Identifiable data will not be destroyed.**

63a **If true, provide rationale for retaining identifiable data indefinitely.**

To input text, click in the light grey area below.

64 Data Access

Click on the check box (or double click and type an "X" if using Google Docs) for each group of individuals that will have access to study data.

If you plan on creating a repository, complete the repository form as well.

☒ **Study Team Members**

☒ **External Collaborators**

☐ **Data coordinating center**

☒ **Sponsor**

☒ **Future Sharing with other researchers**

☐ **Others**

Describe below. To input text, click in the light grey area below.

65 **Describe data sharing plan for each group checked above and state whether researchers plan on sharing identifiable, coded, or de-identified data**

To input text, click in the light grey area below.

Data will only be shared with organizations or individuals outside the study team in a de-identified manner.

Certificate of Confidentiality

Click on the check box (or double click and type an "X" if using Google Docs) to identify each element below that may require additional information to be disclosed in the consent form.

If a Certificate of Confidentiality is not applicable for this study, skip to 68.

66 ☐ **The study requires a Certificate of Confidentiality.**

67 ☐ **NIH has issued a Certificate of Confidentiality for this study.**

68 ☐ **A Certificate of Confidentiality has not been obtained, but there are plans to apply for one.**

COMPENSATION AND COSTS

Compensation

Click on the check box (or double click and type an "X" if using Google Docs).

69 ☒ **Subjects receive compensation.**

70 ☐ **Subject will not receive compensation.**

Skip to question 74 if subjects will not receive compensation.

71 **Total Amount of Compensation**

To input text, click in the light grey area below.

$185

72 **Type of Compensation**

Click on the check box (or double click and type an "X" if using Google Docs) for each form of compensation that will be provided.

☐ Cash ☐ Check ☒ Gift Card

☐ Course Credit ☐ ClinCard ☒ Tango Card

☒ Other

Describe, To input text, click in the light grey area below.

Venmo or PayPal

73 **Proration Schedule**

To input text, click in the light grey area below.

Participants will receive compensation for the three online surveys (within a week of completion of each one separately), and will receive compensation for the number of days they used the APP once every two weeks ($2/day).

74 ☒ **Amount of compensation and its form is reasonable for this population for the activities requested of them.**

75 Costs

Click on the check box (or double click and type an "X" if using Google Docs) each applicable item regarding costs.

☒ **Participants will have no costs associated with this study**

☐ **Standard of care procedures contributing to study data**

☐ **Research procedures not associated with standard of care**

☐ **Administration of drugs / devices**

☐ **Study drugs or devices**

☐ **Transportation and parking**

76 Describe all costs below.

To input text, click in the light grey area below.
